# Supplementary material for: Blood Cultures Time-to-Positivity as an Antibiotic Stewardship Tool in Immunocompromised Children with Gram-Negative Bacteraemia
Source: Antibiotics (Basel). 2025 Aug 21;14(8):847. doi: 10.3390/antibiotics14080847 (PMC12383192; doi:10.3390/antibiotics14080847)
Supplement: Supplementary file 1 [file antibiotics-14-00847-s001.zip › antibiotics-3760101-supplementary.pdf]

Blood cultures time-to-positivity as an antibiotic stewardship tool in immunocompromised children with gram-negative bacteraemia - Supplementary Materials

Table S1. Characteristics of GNB-BSI in paediatric immunocompromised patients who required PICU admission.

TTP (h)	Sex/age	Pathogen	Underlying disease	Infectious focus	Reason for PICU admission	30-day mortality (cause)
6.0	Female / 5y	*E. coli*	HR-ALL, relapse	Unknown	Septic shock	No
6.5	Male / 16y	*E. coli*	HR-ALL, refractory	CRBSI	Septic shock	No
7.2	Male / 6y	*M. morganii*	HR-ALL, HSCT	Unknown	Septic shock	No
8.6	Female / 8y	*E. coli*	HR-ALL, induction	Unknown	Septic shock	No
8.6	Male / 15y	*K. pneumoniae*	Ewing sarcoma	Unknown	Septic shock	No
9.4	Male / 11m	*E. cloacae*	AT/RT	Others	Multidisciplinary management	No
12.0	Male / 3y	*E. cloacae*	AML (M5)	Unknown	Septic shock	No
10.5	Female / 3y	*K. oxytoca*	AML (M7)	Unknown	Septic shock	No
11.5	Male / 11y	*E. coli*	Osteosarcoma	Complicated IAI	Septic shock	No
11.5	Male / 16y	*K. pneumoniae*	AML (M5)	CRBSI	Septic shock	No
12.7	Female / 6y	*K. pneumoniae*	Neuroblastoma	Complicated IAI	Septic shock	No
15.6	Male / 2y	*P. aeruginosa*	HR-ALL, induction	Complicated IAI	Abdominal surgery	No
17.9	Female / 15y	*P. aeruginosa*	HR-ALL, induction	Complicated IAI	Septic shock	No

AML: acute myeloblastic leukaemia; AT/RT: atypical teratoid/rhabdoid tumour; CRBSI: Catheter-related bloodstream infection; HR-ALL: high-risk acute lymphoblastic leukaemia; HSCT: Hematopoietic stem cell transplantation; IAI: intraabdominal infection; PICU: paediatric intensive care unit; TTP: time-to-positivity

Table 2. Characteristics of GNB-BSI in paediatric immunocompromised patients who died in the first 30 days.

TTP (h)	Sex/age	Pathogen	Underlaying disease	Infectious focus	Cause of death
8.1	Male / 4y	*E. coli*	HR-ALL, relapse	Others	Infection + underlying disease
10.8	Male / 5y	*E. coli*	Neuroblastoma	Unknown	Underlying disease
11.3	Male / 8y	*E. coli*	HR-ALL, relapse	Unknown	Underlying disease
10.6	Male / 11y	*E. coli*	HR-ALL, refractory	Unknown	Underlying disease
12.0	Female / 14y	*E. coli*	Neuroblastoma	Unknown	Underlying disease
7.9	Male / 4y	*E. coli*	AML (M3)	Unknown	Underlying disease
7.5	Male / 3m	*E. cloacae*	CMN	Others	Infection

AML: acute myeloblastic leukaemia; CMN: congenital mesoblastic nephroma; HR-ALL: high-risk acute lymphoblastic leukaemia; TTP: time-to-positivity
